# Prone positioning and convalescent plasma therapy in a critically ill pregnant woman with COVID‐19

**DOI:** 10.1002/ccr3.3426

**Published:** 2020-10-26

**Authors:** Massimo Donzelli, Mariachiara Ippolito, Giulia Catalisano, Baldassare Renda, Francesco Tarantino, Orazia Diquattro, Andrea Cortegiani

**Affiliations:** ^1^ Unità Operativa di Anestesiae Rianimazione Azienda Ospedaliera Ospedali Riuniti (AOOR) P.O. Cervello Palermo Italy; ^2^ Department of Surgical, Oncological and Oral Science (Di.Chir.On.S.) University of Palermo Palermo Italy; ^3^ Unità Operativa di Microbiologia Azienda Ospedaliera Ospedali Riuniti (AOOR) Palermo Italy; ^4^ Department of Anesthesia, Intensive Care and Emergency Policlinico Paolo Giaccone University of Palermo Palermo Italy

**Keywords:** case report, COVID‐19, plasma therapy, pregnancy, prone position

## Abstract

Prone positioning is feasible in pregnancy and may have contributed to the positive outcome in this case. Doctors should not be reluctant to move a patient to a prone position just because they are pregnant.

## Graphical abstract

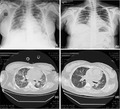

## 1 INTRODUCTION

As the spread of COVID‐19 continues worldwide, a significant number of pregnant women are getting infected and need care. This case describes a 27.4 weeks pregnant woman with critical COVID‐19, treated with protective mechanical ventilation, prone positioning, and hyperimmune plasma. She was discharged with positive maternal and perinatal outcome.

As the number of patients with Coronavirus disease 2019 (COVID‐19) increases worldwide,^1^ many experimental or compassionate interventions have been considered,^2^ but their efficacy remains controversial in most of the cases.^3^ Both prone positioning, already in use in patients with severe acute respiratory distress syndrome (ARDS),^4^, ^5^ and hyperimmune plasma therapy have been proposed for COVID‐19.^6^, ^7^

To date, limited reports have been published on the use of these treatments in pregnant women with COVID‐19.^8^, ^9^ The use of experimental treatments in pregnant women often leads to concerns in both clinicians and researchers.^10^ Nevertheless, COVID‐19 infection in pregnancy leads to hospitalization for a significant proportion of women with 10% requiring respiratory support and this number is expected to increase during the spread of the pandemic,^11^, ^12^ and interventions which have been proven effective are urgently needed. The feasibility of prone positioning in pregnant women has already been described in literature,^13^ in particular during the H1N1 pandemic.^14^ Similarly, specific contraindications against the use of hyperimmune plasma therapy in pregnant women are not reasonably expected.

Although we are still far from definitive confirmations of effectiveness and specific indications, here we report the case of a 34‐year‐old pregnant woman, at 27.4 weeks of gestation, admitted to our emergency department (ED) for critical COVID‐19 and successfully treated in our institution. During her clinical course, clinical decisions were conducted by a multidisciplinary team. The patient was treated with protective ventilation, prone positioning, and hyperimmune plasma therapy, in addition to standard care. The patient was finally discharged home with no sequelae after a total length of stay (LOS) of 43 days and a successful cesarean section (CS) delivery. This case report was prepared following the CARE Guidelines.^15^ The informed consent was obtained from the patient for the presentation of this case.

## 2 CASE PRESENTATION

A 34‐year‐old woman was admitted to the ED of “Ospedali Riuniti Villa Sofia‐Cervello” Palermo, Italy, with dyspnea and tachypnea. The patient was 27.4 weeks pregnant, with no history of previous diseases, no allergies, two previous pregnancies with vaginal births and a previous appendectomy. At the interview, the patient reported a recent international flight and the onset of flu‐like symptoms, with fever, asthenia, and musculoskeletal pain occurring few days after the flight and followed by a rapid clinical worsening. She reported no benefit from paracetamol assumption. Physical examination showed decreased breath sounds to both lungs basis and arterial blood gas (ABG) revealed severe respiratory failure, with SO_2_ 70% and PaO_2_ 38.9 mm Hg in room air (See Table 1).

**Table 1:** Arterial blood gases and laboratory tests results

		pH	PaCO_2_ (mm Hg)	PaO_2_ (mm Hg)	FiO_2_	SO_2_ (%)	HCO_3_^−^ (mmol/L)	Lac (mmol/L)	WBC (×10^3^/µL)	RBC (×10^6^/µL)	Hb (g/dL)	HTC (%)	PLT (×10^3^/µL)	IgG anti SCoV2 (AU/mL)	IgM anti SCoV2 (AU/mL)
HD 0	Arrival at ED	7.439	33.6	38.9	0.21	70.9	23.1	1.1	14	3.5	10.6	32.4	291	NA	NA
HD 0	ICU intubation	7.24	45	183	0.8	98.4	19	0.6	13.3	3.3	9.8	30.5	291	NA	NA
HD 1	Pre‐pronation	7.29	46	58	0.9	87.7	22	1.2	18.1	3.1	9.3	28.8	376	NA	NA
HD 1	Postpronation	7.31	46	121	0.8	98.1	23	1	18.1	3.1	9.3	28.8	376	NA	NA
HD 2	Before HIP administration	7.39	39	112	0.8	97.9	23	1	14.6	2.7	8.3	25.9	380	3.8	2.9
HD 3	After 1st HIP bag	7.35	48	68	0.5	93.7	26	1.2	13.4	2.8	8.7	26.5	397	14.6	10.35
HD 4	After 2nd HIP bag	7.37	45	83	0.6	96.4	25	1	12.2	3.1	9.4	28	387	20.4	14.7
HD 11	Extubation	7.36	41	156	0.5	98.5	23	1.1	8.45	3.3	10.4	32.3	311	65	30
HD 14	Before re‐intubation	7.29	56	83	0.7	95.4	26	0.5	13.7	3.5	10.4	32	233	NA	NA
HD 14	After re‐intubation	7.20	71	123	0.8	97.4	27	0.4	13.7	3.5	9.8	30.3	233	NA	NA
HD 17	C‐section	7.27	68	94	0.5	96.4	25	0.5	8.43	3.4	9.7	30	183	NA	NA
HD 19	Tracheostomy	7.42	40	102	0.4	98	26	0.6	9.26	3.2	9.3	28.7	170	NA	NA
HD 32	ICU discharge	7.40	38	184	0.28	97	23	1.1	4.95	4.0	11.8	36.2	198	NA	NA
HD 42	Hospital discharge	NA	NA	NA	NA	NA	NA	NA	NA	NA	NA	NA	NA	82.6	8.8

Abbreviations: C‐Section, cesarian section; ED, Emergency Department; FiO_2_, inspired fraction of oxygen; Hb, hemoglobin; HCO_3_, Bicarbonate; HD, hospitalization day; HIP, Hyperimmune Plasma; HTC, hematocrit; ICU, Intensive care unit; Lac, lactate; NA, not available; PaCO_2_, arterial partial pressure of carbon dioxide; PaO_2_, arterial partial pressure of Oxygen; PLT, platelets; RBC, red blood count; SCoV2, SARS Corona Virus 2; WBC, white blood count.

Pulmonary embolism was suspected but rapidly excluded with the aid of cardiac ultrasound. Meanwhile, positivity to SARS‐CoV‐2 was detected from nasopharyngeal and oropharyngeal swab specimens, that had been collected at the arrival to the ED and then analyzed. O_2_ therapy was started at 5 L/min with a slight improvement. The patient was transferred to the COVID‐19 pulmonology ward, where O_2_ flow was increased to 15 L/min. Noninvasive ventilation (NIV) was attempted without success, due to poor compliance to the interface (face mask) and no improvements in respiratory failure. The patient was then admitted to the Intensive Care Unit (ICU), where high‐flow nasal cannula (HFNC) oxygen therapy was attempted (FiO_2_ between 80% and 90%; flow 60 lt/min; temperature at 31°C), resulting in a slight improvement of oxygenation, even though tachypnea persisted. During the night, due to the worsening of respiratory failure, the patient was sedated, paralyzed, and intubated and invasive mechanical ventilation was started with protective settings.

On hospitalization day (HD) 1, severe hypoxia persisted (P/F 64.4, SO_2_ 87.7%). Thus, a multidisciplinary consultation was planned, involving neonatologists ad gynecologists. Given the early gestational age, the team decided to continue the pregnancy monitoring during ICU stay, and to perform a cesarean section delivery only in case of a drastic clinical worsening of either the mother or the child. It was also decided to attempt prone positioning, then successfully performed with the use of supports and pads beneath shoulders and hips, to prevent aortocaval compression. The patient started a 12‐hour daily cycle of pronation, and a progressive improvement of oxygenation was registered, allowing a FiO_2_ decrease. The patient underwent a total of four cycles of pronation, lasting 12 hours each. The last cycle was performed on HD 4, without consistent variations of respiratory parameters; therefore, no more cycles were performed.

Meanwhile, the ethics committee (“Comitato Etico Palermo 2”) gave the authorization to start a compassionate treatment with hyperimmune plasma, previously requested. The first hyperimmune plasma bag was administered on HD 2 and the second one after 24 hours, with no complications. IgG and IgM antibodies for SARS‐CoV‐2 increased during the following days (see Table 1).

**FIGURE 1:**
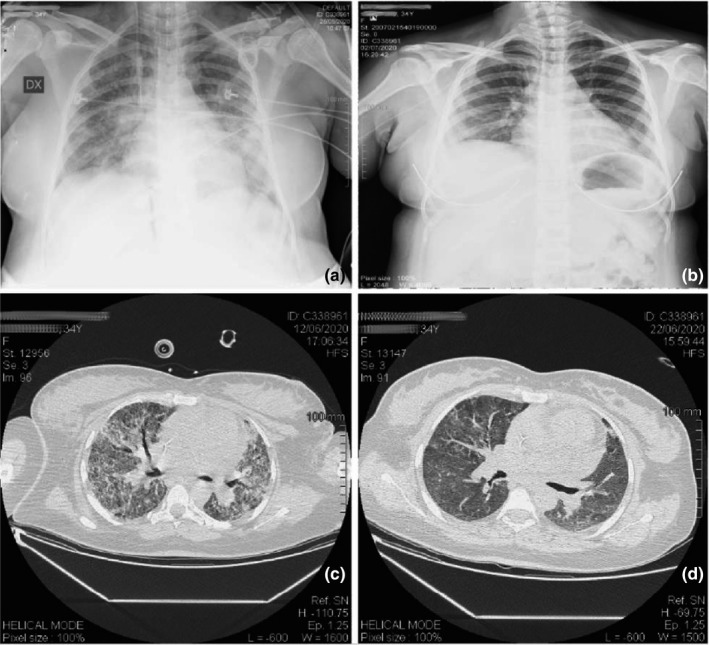
Chest radiographs and Computerized tomographies. The figure shows chest radiographs and chest computerized tomographies (CTs) at different moments. Panel A: Chest radiographs (day 3 from hospital admission) showing multiple bilateral parenchimal opacities tending to confluency, major on the left side. Panel B: Chest radiographs (day 38 from hospital admission) showing residual increase of bronchovascular markings in the basal region, more evident on the right side. Panel C: Chest CT (day 18 from hospital admission) showing multiple bilateral diffuse lung consolidations with ground glass aspect and thickening of lung interstitium. Panel D: Chest CT (day 28 from hospital admission) showing residual consolidations with fibrotic aspect in the upper lobes and diffuse thickening of lungs' interstitium with ground glass prevalent pattern

**FIGURE 2:**
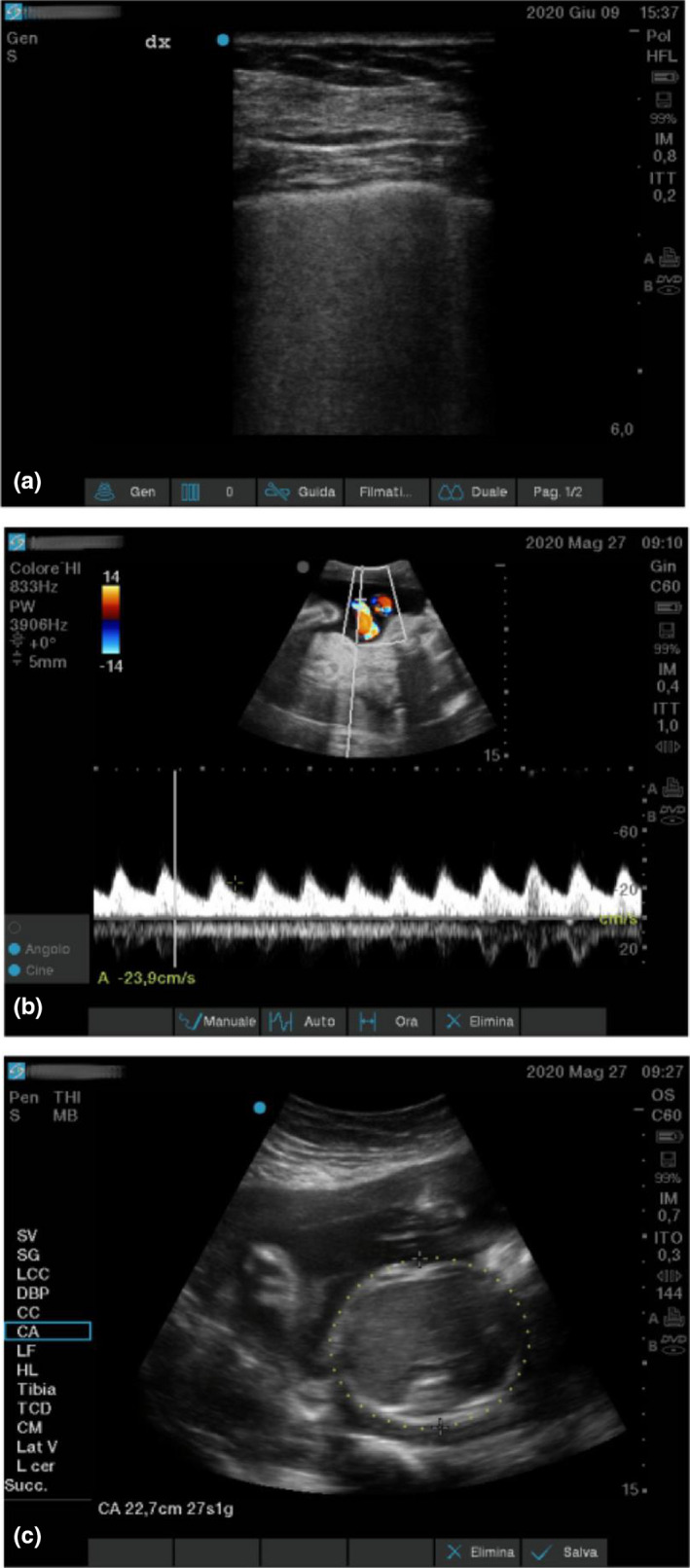
Ultrasounds. Panel A: Lung ultrasound showing multiple confluent B lines and pleural line thickening. Panel B: Fetal monitoring through umbilical artery doppler. Panel C: Fetal monitoring through biometrical measurements, for example, head circumference: showing regular fetal development

During ICU stay, the best level of positive end expiratory pressure (PEEP) was evaluated and set daily through PEEP trials. Chest radiographs had revealed bilateral multiple parenchymal opacities, worse on the left lung (See Figure 1A), then partially improved (See Figure 1B). Lung ultrasounds were used for daily monitoring, to reduce the pregnant patient's exposure to ionizing radiations. Lung ultrasounds showed multiple confluent B lines and thickening of the pleura (See Figure 2A). During the entire ICU stay, the patient was visited daily by gynecologists and obstetricians in order to monitor the advancement of the pregnancy and the status of the fetus (see Figure 2B,C). Fetal well‐being was monitored using ultrasound evaluation of active movements, amniotic fluid, and umbilical artery Doppler. Maternal and fetal monitoring was regularly performed both in the supine and in the prone position.

Pharmacological therapy consisted in low molecular weight heparin (enoxaparin sodium 4000 UI twice a day) to prevent thromboembolic events, betamethasone (12 mg daily for 2 days) to accelerate fetal lung maturity and empirical antibiotic therapy with clarithromycin (500 mg twice a day) and ceftriaxone (2 g daily), then substituted by vancomycin (2 g daily) because of a bronchial aspirate positive to *Staphylococcus aureus,* followed also by positive hemocultures. According to the local protocol, patient's bronchial aspirate was weekly tested for common respiratory pathogens during ICU stay.

On HD 7 weaning procedures started, neuromuscular blocking agents were stopped, sedation minimized and respiratory support gradually reduced. Progressive weaning led to extubation on HD 11; thereby, the patient was assisted with alternated cycles of HFNC oxygen therapy and conventional O_2_ therapy. The patient was increasingly tachypnoic when on the night of HD 14, she was reintubated. On the same day, the antibiotic therapy was modified, because of a bronchial aspirate positive for *Pseudomonas aeruginosa* sensitive to Meropenem (1 g × 3). The following day the patient's bronchial aspirate came back negative for SARS‐CoV‐2.

On HD 17, at 30.1 weeks of pregnancy, due to reduced pulmonary compliance, and a worsening of hypoxia and hypercapnia, it was decided to perform a cesarean section and deliver the fetus. At birth, the infant was hypotonic, hyporeactive without autonomous respiratory activity (APGAR: 1′:3, after intubation 5′:6; 10′:7), and was therefore intubated and transferred to neonatal intensive care unit (NICU). The newborn's bronchial aspirate tested negative for SARS‐CoV‐2. On HD 18, a successful extubation was performed on the newborn, followed by Nasal Continuous Positive Airway Pressure (nCPAP) support and the start of oral feeding.

The mother's postoperative course proceeded without complications. Chest computerized tomography (CT) was performed after the delivery, showing multiple, diffuse, bilateral parenchymal lung thickening with consolidations and a ground glass pattern with thickening of pulmonary interstitium. (See Figure 1C).

On HD 19, the patient's condition remained basically unchanged, so in order to guarantee better gas exchange and promote weaning from mechanical ventilation, a percutaneous tracheostomy was performed under bronchoscopy sight, without complications.

Then, sedation was reduced and weaning attempted, with a full restoration of valid spontaneous ventilation on HD 23.

General conditions improved, the patient started oral feeding together with passive and active physiotherapy. Chest CT also showed a reduction of pulmonary consolidations. (See Figure 1D).

On HD 32, the patient was transferred to the pneumology ward in satisfactory general conditions. A few days later, tracheostomy cannula was removed. The patient was transferred to the infectious disease ward on HD 39 and ultimately discharged from hospital on HD 42. The patient is still undergoing periodical clinical and radiological follow‐up.

## 3 DISCUSSION

This case described the successful maternal and perinatal clinical course of a pregnant patient with critical COVID‐19 after 43 days of hospitalization, including 33 days of ICU stay. The most relevant interventions were prone positioning and hyperimmune plasma therapy.

Our findings do not prove the efficacy of the interventions, also considering the lack of high‐quality evidence in this specific setting (maternal/perinatal). Moreover, many clinical choices may have been influenced by the specific context, that is, a nonoverwhelmed hospital, temporarily intended for the care of COVID‐19 patients only. This may further limit the external validity of the described findings. Also, the decision not to perform CT scan is in contrast with the evidence that chest CT has become the reference imaging method for the diagnosis of COVID‐19 pneumonia and treatment follow‐up. Despite the pregnant women's exposure to CT radiation barely reaches the dose that causes harm to the fetus, we preferred a more conservative approach. However, our case suggests the feasibility of prone positioning in pregnant COVID‐19 patients and provides useful insights on the pivotal role of multidisciplinary debates on clinical choices, in the context of lack of evidence. As an example, it was collegially decided to perform preterm cesarean section only if a drastic worsening of the patient's condition occurred.

To date, only limited evidence is available on pregnant patients with COVID‐19 and newborns from infected mothers.^16^, ^17^, ^18^ Data regarding these specific populations would be of great interest, considering the physiological changes in pregnancy compared to nonpregnant women. In the case of mainly respiratory diseases, such as COVID‐19 or previously pandemic viral diseases (eg, H1N1), it is important to consider that a pregnant woman has an increased oxygen uptake and a high basal metabolism, a reduced functional residual capacity, an elevated diaphragm and airway edema, potentially contributing to worsen the clinical condition.^19^ Furthermore, the hypercoagulable status, typical of pregnancy, may also have a role in deteriorating COVID‐19 into its severe or critical forms in these patients.^20^ No clear evidence exists on the determinants of severity in the context of COVID‐19, and the clinical course is even less predictable in pregnant women, as an under‐studied population.

Prone positioning has been proven to reduce mortality in ARDS,^5^ improving gas exchange and ventilation/perfusion ratio, through the recruitment of previously nonaerated areas of the lung. Only limited evidence exists on prone position in pregnant women. Among the concerns related to prone positioning in pregnant patients, aortocaval compression can occur, causing severe hypotension, that must be avoided. Thus, the procedure becomes even more demanding for nurses, and fetal monitoring is also difficult once the patient is prone positioned.^9^ On the other hand, the prone position has been described as safe and potentially advantageous in healthy or pre‐eclamptic pregnant patients,^21^, ^22^ and recently described in pregnant patients with COVID‐19.^17^, ^23^, ^24^ Despite encouraging, data on COVID‐19 patients, both pregnant and nonpregnant, remain limited.

No clear evidence exists on convalescent plasma effectiveness in reducing the risk of death or the need for respiratory support in patients with COVID‐19, and limited data are available on its safety.^7^ We registered an increase in the patient's antibody title after administration of hyperimmune plasma, as documented by immunoglobulin (Ig) levels (Table 1). Nevertheless, the presence of antibodies against SARS‐CoV‐2 does not directly indicate protective immunity, since direct associations have not yet been proved,^25^ and no conclusions can be drawn from our case on the topic.

The procedure of tracheostomizing a critically ill patient is often a source of debate for the clinical team, especially on patient selection, timing, best setting, and methods. In the context of COVID‐19 pandemic, new concerns regard healthcare workers' exposure to a high risk of intraprocedural contagion. A conservative approach is suggested, together with adequate protective personal equipment and appropriate training provided to the team ^26^, ^27^, ^28^ and this approach was successfully followed in our case.

Most of the proposed pharmacological therapies for COVID‐19 are still controversial,^3^, ^29^, ^30^ and none of them was administered in this case, except for empirical antibiotics, used with the purpose of superinfections prophylaxis. Despite the emerging evidence on the beneficial role of corticosteroids (eg, dexamethasone) in COVID‐19,^31^ no conclusions can be drawn from this case on the use of betamethasone, administered with the purpose of preventing newborn respiratory distress syndrome, as the need of a preterm delivery was anticipated.

The negativity of the newborn infant to SARS‐CoV‐2 testing reflects what has already been described in available literature, reporting rare vertical transmissions.^17^, ^32^

We believe that collective decisions, such as those regarding prone positioning, timing of cesarean section and tracheostomy, positively influenced the outcome of both the mother and the newborn. Similarly, we believe that the close co‐operation with gynecologists and neonatologists, performing daily visits to the patient and fetal monitoring during the ICU stay, had relevant impact on the case. It can be argued that a multidisciplinary management of COVID‐19 critically ill pregnant women is needed to ensure the best maternal and fetal care.

## CONFLICT OF INTEREST

AC is an Associate Editor for Clinical Case Reports. All the other authors declare no conflicts of interest.

## AUTHORS' CONTRIBUTION

MD: treated the patient, conceived the content, collected the data, and drafted the manuscript. MI, GC, and AC: conceived the content and drafted the manuscript. BR, FT, and OD: treated the patient and revised the manuscript critically for important intellectual contents. All the authors gave the final approval of the version to be published and agreed to be accountable for all aspects of the work in ensuring that questions related to the accuracy or integrity of any part of the work are appropriately investigated and resolved.

## ETHICAL APPROVAL

The informed consent was obtained from the patient for the presentation of this case.
